# Release Behavior and Toxicity Profiles towards Leukemia (WEHI-3B) Cell Lines of 6-Mercaptopurine-PEG-Coated Magnetite Nanoparticles Delivery System

**DOI:** 10.1155/2014/972501

**Published:** 2014-05-08

**Authors:** Dena Dorniani, Aminu Umar Kura, Samer Hasan Hussein-Al-Ali, Mohd Zobir bin Hussein, Sharida Fakurazi, Abdul Halim Shaari, Zalinah Ahmad

**Affiliations:** ^1^Materials Synthesis and Characterization Laboratory (MSCL), Institute of Advanced Technology (ITMA), Universiti Putra Malaysia, 43400 Serdang, Selangor, Malaysia; ^2^Vaccines and Immunotherapeutics Laboratory (IBS), Universiti Putra Malaysia, 43400 Serdang, Selangor, Malaysia; ^3^Laboratory of Molecular Biomedicine, Institute of Bioscience, Universiti Putra Malaysia, 43400 Serdang, Selangor, Malaysia; ^4^Faculty of Pharmacy, Isra University, P.O. Box 22, Amman 11622, Jordan; ^5^Physics Department, Faculty of Science, Universiti Putra Malaysia, 43400 Serdang, Selangor, Malaysia; ^6^Chemical Pathology Unit, Department of Pathology, Faculty of Medicine and Health Sciences, Universiti Putra Malaysia, 43400 Serdang, Selangor, Malaysia

## Abstract

The coating of an active drug, 6-mercaptopurine, into the iron oxide nanoparticles-polyethylene glycol (FNPs-PEG) in order to form a new nanocomposite, FPEGMP-2, was accomplished using coprecipitation technique. The resulting nanosized with a narrow size distribution magnetic polymeric particles show the superparamagnetic properties with 38.6 emu/g saturation magnetization at room temperature. Fourier transform infrared spectroscopy and the thermal analysis study supported the formation of the nanocomposite and the enhancement of thermal stability in the resulting nanocomposite comparing with its counterpart in free state. The loading of 6-mercaptopurine (MP) in the FPEGMP-2 nanocomposite was estimated to be about 5.6% and the kinetic experimental data properly correlated with the pseudo-second order model. Also, the release of MP from the FPEGMP-2 nanocomposite shows the sustained release manner which is remarkably lower in phosphate buffered solution at pH 7.4 than pH 4.8, due to different release mechanism. The maximum percentage release of MP from the nanocomposite reached about 60% and 97% within about 92 and 74 hours when exposed to pH 7.4 and 4.8, respectively.

## 1. Introduction

Leukemia is a type of cancer of the blood or bone marrow which can affect people at any age and the rate of cure can be depending on the age of patient as well as the types of leukemia. Leukemia can be distinguished via an abnormal proliferation and accumulation of immature white blood cells which are called blasts. The drug, 6-mercaptopurine (MP), is one of anticancer drugs that belong to the class of antimetabolites which can be used to treat different types of diseases such as inflammatory bowel disease, pediatric non-Hodgkin's lymphoma, and leukemia [1]. Nowadays, iron oxide nanoparticles (FNPs) and their nanocomposites have found an increasing attention in biomedical application due to their unique physicochemical properties, such as surface-coat ability, superparamagnetism, nontoxicity, high chemical stability, and high-level accumulation in the target area [2–6].

Because of strong magnetic dipole-dipole attraction between the particles in Fe_3_O_4_, some stabilizers such as surfactants or polymeric compounds [7] with specific functional groups have been used in order to prevent the aggregation and modify the surface of iron oxide nanoparticles [8]. The desirable properties of magnetic nanoparticles such as high surface area, uniform size, biocompatibility and high superparamagnetic with tailor-make properties, result in better demand of this type of nanoparticles for bioapplications [9, 10]. It is worth noting that cationic nanoparticles, including gold and polystyrene, can cause hemolysis and blood clotting. On the other hand, generally anionic particles are quite nontoxic [11].

The drug can be either attached or loaded to the surface of superparamagnetic nanoparticles or embedded in the carrier matrix. Through the blood circulation, it can be delivered to the desired tumor site by means of an external localized magnetic field gradient [12]. Therefore, with magnetic drug targeting (MDT) system [13, 14] drugs can be reached and released into the target tumor site [15]. Therefore, in order to have the effective release, biodegradable and biocompatible nanoparticle formulations are desired. To increase the stability of iron oxide nanoparticles in colloidal suspension and modification of the surface, various biocompatible and biodegradable polymers such as polyethylene glycol (PEG) [16–19], polyvinylpyrrolidone (PVP) [2], polyvinyl alcohol (PVA) [16, 20, 21], and natural polymers like chitosan [22–25] and dextran [26, 27] can be employed.

The surface coating of iron oxide nanoparticles with PEG chains can be used to decrease the reticuloendothelial system (RES) clearance, toxicity, and enzymatic degradation and also increase water solubility, stability of nanoparticles, and prolonged presence in the circulation half-life* in vivo* [15, 19]. In addition, Food and Drug Administration (FDA) has approved the use of polyethylene glycol for human intravenous, oral, and dermal applications [15].

In the present work, we have selected 6-mercaptopurine as a model drug to be loaded into the surface of magnetite nanoparticles, precoated using polyethylene glycol (PEG) as a stabilizer and size controlling agent. The main objective of this work was to explore the potential use of iron oxide nanoparticles (FNPs) coated with PEG as a starting material for the formation of a new nanocomposite. Optimization was done by using two different concentrations of 6-mercaptopurine, 0.5% and 2% (w/w), containing the same amounts of PEG. The effect on viability of leukemia cell lines (WEHI-3) when exposed to these compounds (FPEGMP-0.5 and FPEGMP-2) was examined. The resulting optimized nanocomposite (FPEGMP-2) was then used as a controlled-release formulation of active drug, MP.

## 2. Materials and Methods

### 2.1. Materials

Analytical grade chemicals were used in this work without further purification. Chemicals used for the synthesis of iron oxide nanoparticles were ferrous chloride tetrahydrate (FeCl_2_·4H_2_O ≥ 99%, Merck KGaA, Darmstadt,   Germany), ferric chloride hexahydrate (FeCl_3_·6H_2_O, 99%, Merck, KGaA, Darmstadt, Germany), and ammonia solution (25%) from Scharlau. For coating of iron oxide with polymer, polyethylene glycol with average M.W. 300 was used, purchased as a raw material from Acros Organics BVBA. 6-Mercaptopurine monohydrate with 99.5% purity was purchased from Acros Organics (Fair Lawn, NJ, USA). Dimethyl sulfoxide (DMSO) was supplied by Ajax Finechem (Sydney, Australia) and distilled deionized water (18.2 M·Ωcm^−1^) was used throughout the experiments.

### 2.2. Preparation of Magnetite Nanoparticles

To synthesize iron oxide nanoparticles, a mixture of 2.43 g ferrous chloride tetrahydrate, 0.99 g ferric chloride hexahydrate, and 80 mL of distilled deionized water in the presence of 6 mL of ammonia hydroxide (25% by mass) was exposed to ultrasonic irradiation for around 1 hour as previously reported by Lee and coworkers [28]. Then the precipitates were centrifuged and washed for 3 times to remove all impurities, washed and dispersed into 100 mL distilled deionized water, and mixed by 2% PEG. The mixture was stirred for 24 hours and the resulting black precipitates were collected by a permanent magnet, washed for 3 times to remove the excess polymer (PEG) which is not participated in the coating process, and then dried in an oven. The 2% of drug solution, 6-mercaptopurine, which was dissolved in dimethyl sulfoxide, was added to the magnetite-PEG and the mixture was stirred for 24 h. Finally, the coated magnetite was washed for three times and dried in an oven. In addition, to optimize the percentage of drug loading, two different percentages (0.5% and 2%) were prepared using the same amount of PEG (2%) under the same conditions. We compared the two nanocomposites (FPEGMP-0.5 and FPEGMP-2) in terms of their cytotoxic effect on antileukemic cancer cell lines.

## 3. Cell Viability Study

### 3.1. Cell Culture

A mouse myelomonocytic leukemic cell line, WEHI-3B, was obtained from American Type Culture Collection (Manassas, VA, USA). The cells were cultured in DMEM medium (Dulbecco's Modified Eagle Medium, Gibco) supplemented with 10% heat inactivated foetal bovine serum (FBS) and 1% antibiotics (100 units/mL penicillin/100 mg/mL streptomycin). Cells were grown in a humidified incubator at 37°C (95% room air, 5% CO_2_) and used for seeding and treatment after reaching 90% confluence. The media were changed after two days and subculture was done between 3 and 5 days throughout the experiment.

Cells were seeded at 1 × 10^5^ cells/mL into 96 well plates and left overnight in a CO_2_ incubator to get attached. The coated or uncoated nanoparticles, pure 6-mercaptopurine, were dispersed in DMEM medium and 100 µL final volume per concentration was added to each well. A stock solution of 10 mg/mL from each nanoparticles and pure 6-mercaptopurine was prepared in media and subsequently diluted to obtain the desired concentration of 1.87–60 *μ*g/mL. Wells containing cells and media only were used as control.

### 3.2. Cytotoxicity Testing

The cytotoxicity and anticancer effects of the drugs on the cells were measured using MTT (SIGMA) proliferation assay. In brief 20 µL of MTT solution (5 mg/mL in phosphate buffered saline) was added to each well and left in the incubator at 37°C for 2 hours. The medium containing MTT was removed gently and replaced with dimethyl sulfoxide (DMSO) 100 *μ*L/well. This is to dissolve the blue crystals formed due to the reduction of tetrazolium by living cells. Absorbance at 570 nm and 630 nm (background) was measured using a microplate enzyme-linked immunosorbent assay reader (ELx800, BioTek Instruments, Winooski, VT, USA). All experiments were carried out in triplicate and the results are presented as the mean ± standard deviation:

(1) $$\begin{array}{c} \text{Cell viability}\left(\%\right)=\frac{\text{[Average] test}}{\text{[Average] control}}\times 100. \end{array}$$

### 3.3. Controlled-Release Study

In order to study the drug release profiles of 6-mercaptopurine (MP) from FPEGMP-2 nanocomposite, two pH levels (7.4 and 4.8) were used at 25°C due to the similarity to the pH of blood and that of stomach, respectively [1, 18, 29–31]. About 10 mg of FPEGMP-2 nanocomposites was added to the mixture of 1 mL HCL and 3 mL HNO_3_ and marked it up to 25 mL by distilled deionized water and stirred for around 1 h. Due to the observed intense absorbance at 330 nm in the UV-Vis spectrum, the accumulated release amount of MP from FPEGMP-2 nanocomposite was measured at *λ*_max⁡_ = 330 nm. It is obvious that phosphate buffered solution contains different anions such as Cl^−^, HPO_4_^2−^, and H_2_PO_4_^−^, which can affect the rate of the release.

## 4. Characterization

Powder X-ray diffraction patterns were obtained in a range of 5–70° using a Shimadzu diffractometer, XRD-6000 (Tokyo, Japan), instrument to determine the crystal structure of the samples using CuK_*α*_ radiation (*λ* = 1.5406 Å) at 40 kV and 30 mA. Fourier transform infrared spectra of the materials were recorded over the range of 400–4000 cm^−1^ on a Thermo Nicolet FTIR (AEM, Madison, WI, USA) with 4 cm^−1^ resolution, using the KBr disc method with approximately 1% of the sample in 200 mg of spectroscopic grade potassium bromide, and the pellets were pressed at 10 tons. Thermogravimetric and differential thermogravimetric analyses (TGA-DTG) were performed using a Mettler-Toledo instrument (Greifensee, Switzerland) in 150 *μ*L alumina crucibles in the range of 20–1000°C at a heating rate of 10°C/min. In order to observe the morphology, average particle size, and size distribution of iron oxide and FPEGMP-2 nanocomposite, transmission electron microscopy (Hitachi, H-7100 at an accelerating voltage of 100 kV) was used. An ultraviolet-visible spectrophotometer (Shimadzu 1650 series, Tokyo, Japan) was used to determine the optical and controlled-release properties of MP from the FPEGMP-2 nanocomposite.

## 5. Results and Discussion

### 5.1. Powder X-Ray Diffraction

**Figure 1:**
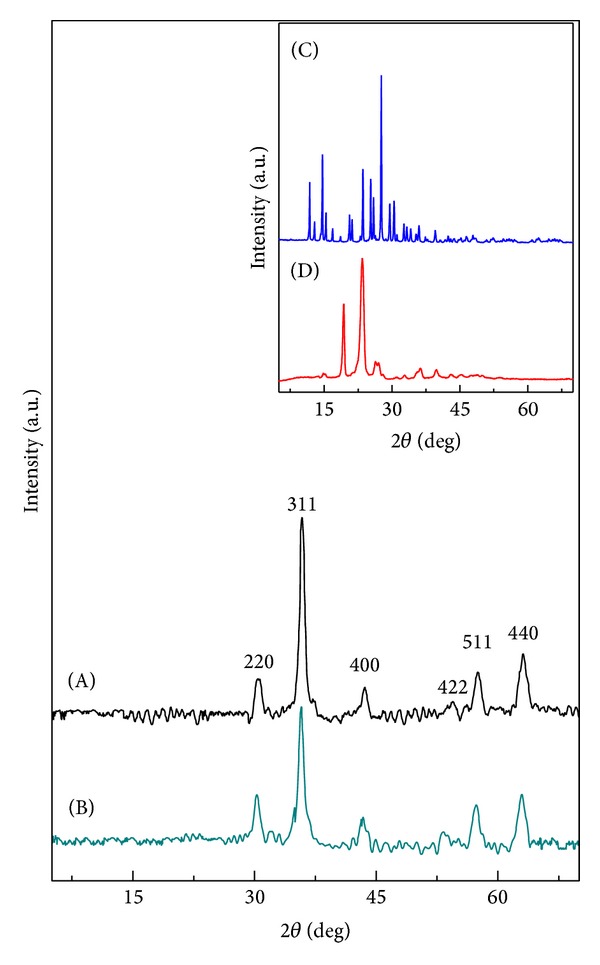
XRD patterns of FNPs (A) and FPEGMP-2 nanocomposite (B). The inset shows the XRD patterns of pure MP (C) and pure PEG (D).

The X-ray diffraction patterns of the naked magnetite iron oxide nanoparticles (FNPs) and iron oxide nanoparticles coated with polyethylene glycol and 6-mercaptopurine (FPEGMP-2) are shown in Figure 1. The inset of Figure 1 shows the XRD patterns of pure 6-mercaptopurine (MP) and the polyethylene glycol (PEG). The two main diffraction peaks revealed at 2*θ* = 10.5° and 20.6° in Figure 1(C) were the characteristic diffraction peaks of pure PEG [32]. The diffraction pattern of pure MP shows many intense sharp peaks in the fingerprint region, indicating the crystalline nature of MP that can be observed at 2*θ* = 11.8°, 14.6°, 16.8°, 21.2°, 23.5°, 25.3°, 25.9°, 27.5°, 29.5°, and 30.3° (Figure 1(D)) [1].

Six characteristic peaks can be observed in FNPs and FPEGMP-2 nanocomposite which were marked by their indices (220), (311), (400), (422), (511), and (440) Bragg reflection, appeared at 2*θ* = 30.1, 35.9, 43.3, 54.2, 57.8, and 63.2, respectively. These peaks confirm that the resultant FNPs was pure magnetite Fe_3_O_4_ with a cubic inverse spinal structure [33, 34]. Due to the absence of the characteristic superlattice diffractions at (210), (213), and (300), it can be confirmed that there is no coexistence of maghemite (*γ*-Fe_2_O_3_) phase in both iron oxide nanoparticles and FPEGMP-2 nanocomposite [35, 36]. Moreover, the result shows that the coating process and the modification of iron oxide nanoparticles after coating with polymer and drug (PEG-MP) did not result in any phase change of the crystal structure of magnetite iron oxide nanoparticles [3, 15, 37]. Using the Debye-Sherrer equation (*D* = *Kλ*/*β*cos⁡*θ*), the average crystallite size of the FNPs was calculated using the (311) XRD pattern, resulting in a value of about 3 nm [33].

### 5.2. Infrared Spectroscopy (FTIR)

**Figure 2:**
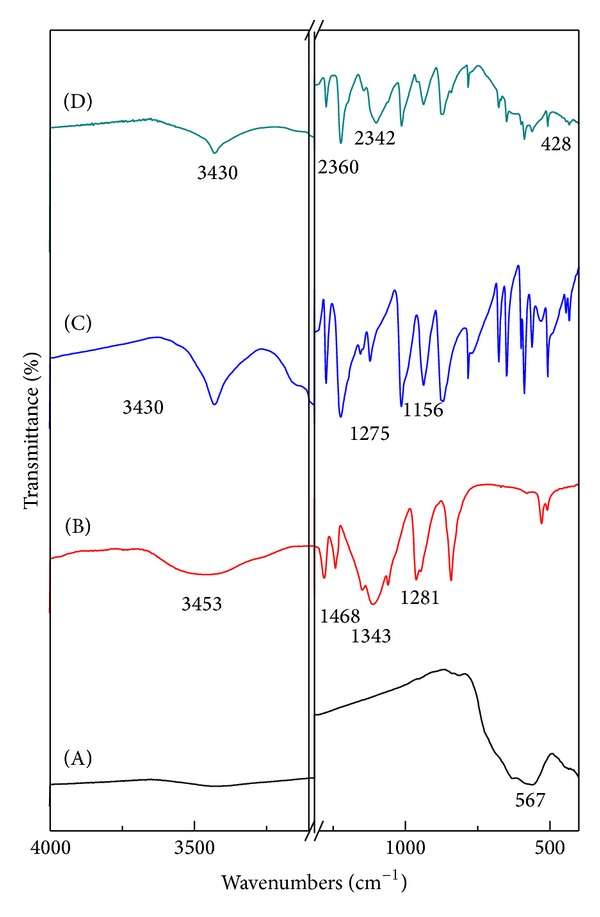
FTIR spectra of FNPs (A), pure PEG (B), pure MP (C), and FPEGMP-2 nanocomposite (D).

In order to realize the attachment of polymer (PEG) to the magnetite nanoparticles and the mechanism of binding, infrared spectroscopic technique was used. Fourier transform infrared (FTIR) spectra for the iron oxide nanoparticles (FNPs), pure polyethylene glycol (PEG), pure 6-mercaptopurine (MP), and iron oxide nanoparticles coated with PEG and MP (FPEGMP-2) are shown in Figure 2. In case of naked iron oxide nanoparticles, a band observed at 567 cm^−1^ is assigned to stretching vibration of Fe–O in Fe_3_O_4_ which is shifted to lower wavenumber, 428 cm^−1^, due to the *ν*_Fe−S_ and *ν*_Fe−N_ vibration modes (Figure 2(A) and (D)) [1, 22]. In Figure 2(B), the main characteristic absorption bands appearing at 2889 cm^−1^ can be assigned to C−H stretching vibration and another two bands at 1468 cm^−1^ and 1343 cm^−1^ belong to the C−H bending vibration. In addition, two characteristic bands at 1281 cm^−1^ and 1094 cm^−1^can be assigned to the O−H and C−O−H stretching vibration, respectively [38].

The absence of a band at 1156 cm^−1^ which belongs to the (*ν*_C=S_ /ring vibration) confirms the participation of an exocyclic (S) atom in metallic bonding of the heterocyclic ligand in the Fe(II) coordination compound (Figure 2(D)) [39]. In addition, the absence of the characteristic absorption band at 1275 cm^−1^ (C=S group) in the FPEGMP-2 nanocomposite, compared to pure 6-mercaptopurine, confirmed the formation of the 6-mercaptopurine complex by the sulfur atom (Figure 2(D)) [39]. An absorption band at 428 cm^−1^ in the FPEGMP-2 nanocomposite proved the presence of magnetite nanoparticles after coating procedure. Therefore, this clearly indicates that the iron oxide nanoparticles were successfully coated with PEG and MP.

### 5.3. Thermal Analysis

**Figure 3:**
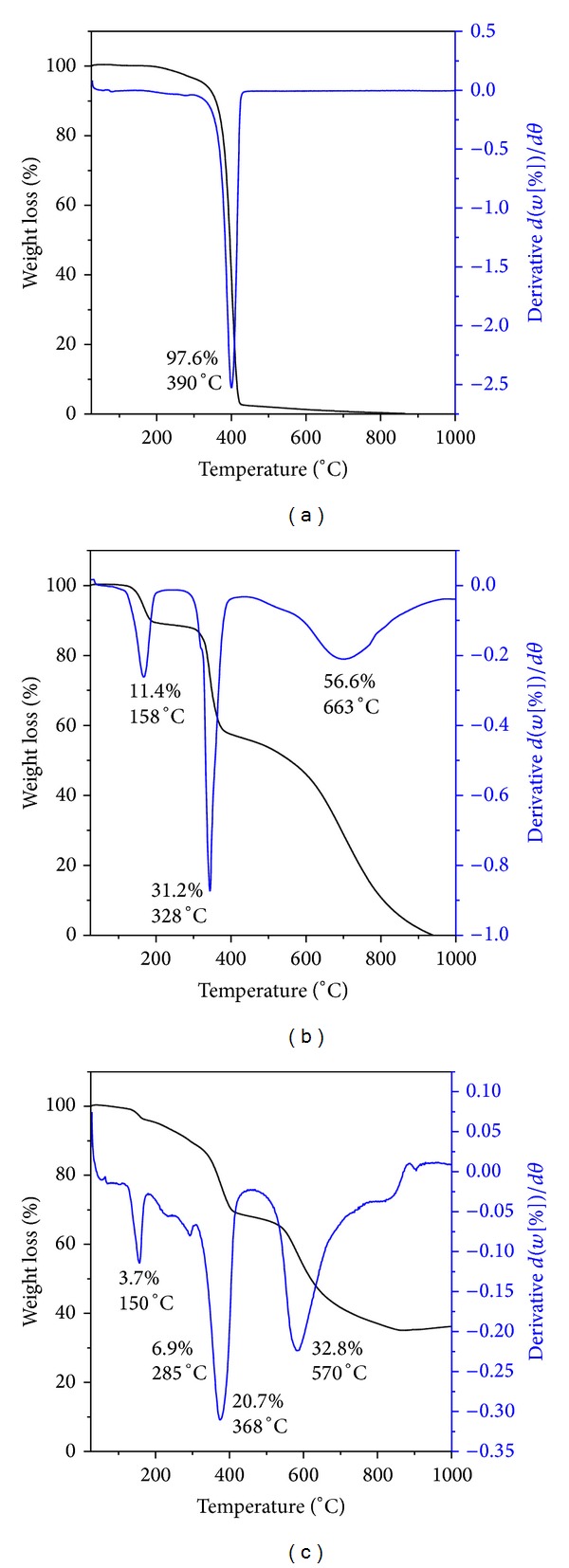
TGA of (a) pure PEG, (b) pure MP, and (c) FPEGMP-2 nanocomposite.

In order to study the physical changes in the materials, thermogravimetric and differential thermogravimetric analyses (TGA-DTG) were used. Due to the molecular structure of the sample and different physicochemical reactions, the thermogram data can be changed. The thermal behavior of the pure PEG, pure MP, and FPEGMP-2 nanocomposite obtained by TGA-DTG analyses is shown in Figure 3. The thermogram for the pure polymer (PEG) shows a sharp maximum temperature at 433°C with 97.6% weight loss. The TGA curves of free MP (Figure 3(b)) show three stages of weight loss over the temperature range from 25°C to 1000°C. The crystalline water was removed at 158°C with a total weight loss of 11%. The second stage shows the sharp mass reduction at temperature maxima of 328°C with the weight losses of 31.2%, presumably due to the decomposition of 6-mercaptopurine which agrees well with the previous study. The mass fragmentation and the thermal decomposition process are not exactly the same; therefore, the weight loss observed may be due to the loss of an HCS group at this step. The third stage was followed at 663°C with the weight losses of 56.6% [40].

The FPEGMP-2 nanocomposite (Figure 3(c)) shows the mass reduction starting from 43°C and completed at 940°C with four-weight losses (43–170°C, 3.7%; 185–334°C, 6.9%; 329–504°C, 20.7%; and finally 522–940°C, 32.8%). The first stage of weight loss might be due to the removal of adsorbed water. The onsets of decomposition of free MP, FNPs, and uncoated PEG was observed between 185 and 334°C. A sharp peak in the region of 329–504°C might be due to the decomposition of PEG coated with MP, free drug MP, and FPEGMP-2 nanocomposite. Finally, the last stage was observed in the region of 522–910°C which may be due to the decomposition of free drug and FPEGMP-2 nanocomposite. Therefore, due to the coating process the thermal stability of MP in FPEGMP-2 nanocomposite was enhanced.

### 5.4. Magnetic Properties

**Figure 4:**
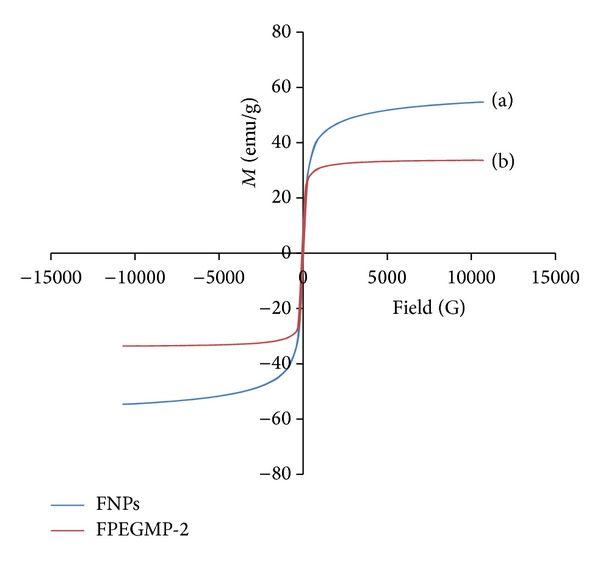
Magnetization plots of (a) FNPs and (b) FPEGMP-2 nanocomposite.

Superparamagnetic property is required for magnetic targeting carriers and biomedical applications [15]; therefore, the magnetic performance of the FNPs (Figure 4(a)) and FPEGMP-2 (Figure 4(b)) was determined using a vibrating sample magnetometer at room temperature. As can be observed, the saturation magnetization of magnetite nanoparticles was about 54.64 emu/g compared to 33.62 for FPEGMP-2 nanocomposite, which is in good agreement with previous works [15, 36, 41, 42]. The decrease of saturation magnetization was only due to the existence of coated materials on the surface of magnetite nanoparticles, which causes the exchange of electrons between the surface of Fe atoms and the PEG polymers [8, 43]. Due to the method of synthesis and the particle size, the saturation magnetization of bare iron oxide can be changed. Therefore, the value of saturation magnetization is usually lower than the theoretical value expected [44–46].

The magnetization curves show narrow hysteresis for both samples, revealing that they were soft magnets with superparamagnetic properties [47].  Table 1 listed the saturation magnetization (*M*_*s*_), remanent magnetization (*M*_*r*_), and coercivity (*H*_*c*_) values which were obtained from the magnetization curves. Due to a good magnetism property (high saturation magnetization) even after coating procedure, the FPEGMP-2 nanocomposite can be easily separated with the help of the external magnetic field [34]; therefore, FPEGMP-2 can be used in biomedical applications.

### 5.5. Particle Size and Size Distribution Properties

**Figure 5:**
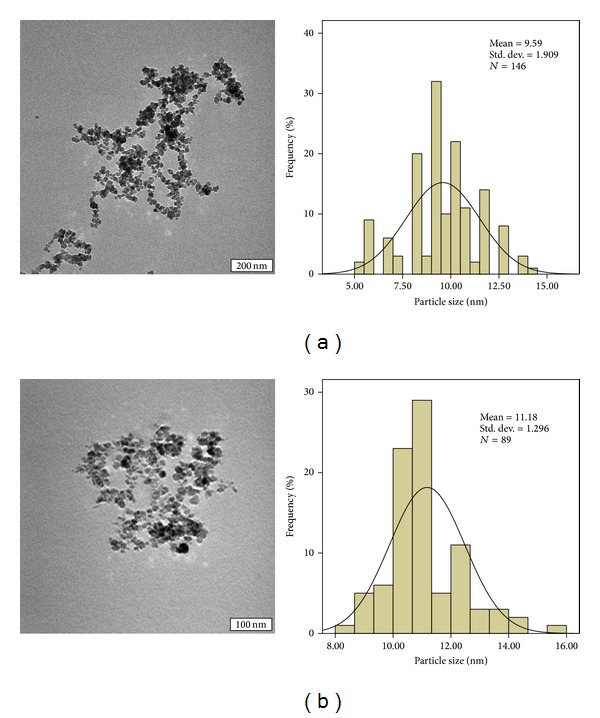
TEM micrographs of (a) iron oxide magnetite nanoparticles with 200 nm microbar and the particle size distribution, (b) FPEGMP-2 nanocomposite with 100 nm microbar and the particle size distribution.

Figure 5 shows the size and shape of the naked FNPs and FPEGMP-2 nanocomposite. The particle size distribution was determined by measuring the diameters of around 100 nanoparticles randomly through the TEM images and using a UTHSCSA ImageTool software. It can be observed that the nanoparticles are well-dispersed and uniform in size and shape although some agglomerate clusters exist due to the magnetization effect [15]. Figures 5(a) and 5(b) show that the pristine FNPs and FPEGMP-2 nanocomposite were nearly spherical in shape and were essentially monodisperse. The average size of FNPs before and after coating is generally similar, around 10 ± 2 nm and 11 ± 1 nm, respectively. From such small differences in the size of FNPs and FPEGMP-2 nanocomposite it can be found that the PEG-MP was successfully coated on the surface of magnetite nanoparticles [3, 48].

### 5.6. Release Study of MP

**Figure 6:**
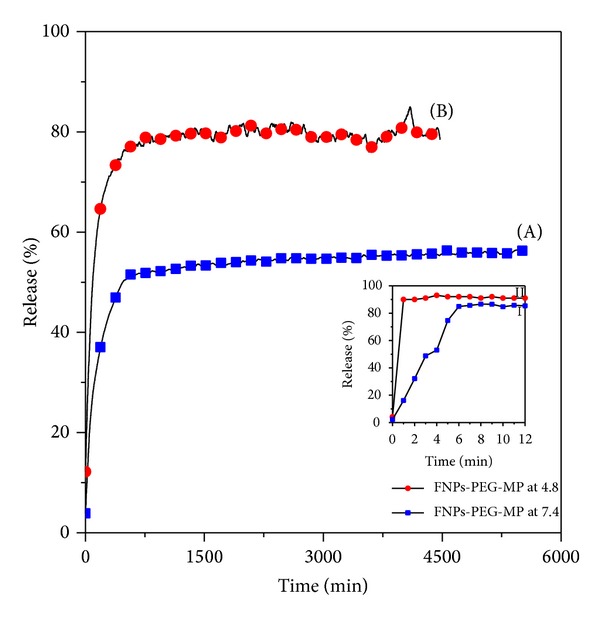
Release profiles of MP from the FPEGMP-2 nanocomposite into (A) phosphate buffered solution at pH 7.4 and (B) phosphate buffered solution at pH 4.8. The inset shows the release profiles of MP from its physical mixture of FNPs-PEG-MP into phosphate buffered solution (I) at pH 7.4 and (II) at pH 4.8.

Through a UV spectrophotometer and a calibration curve equation the percentage of MP loading into FPEGMP-2 nanocomposite was measured to be around 5.6%. The cumulative release profiles of MP from the FPEGMP-2 nanocomposites were investigated by adding the FPEGMP-2 nanocomposite into phosphate buffered solutions at pH 7.4 and 4.8.  Figure 6 shows the release profiles of MP from the abovementioned nanocomposite and the inset shows the MP release from a physical mixture of MP with Fe_3_O_4_-PEG into the same solutions. The release of MP from the physical mixture was found to be very fast, 4 and 7 minutes at pH 4.8 and 7.4, respectively. This indicates that the release of MP is not in the sustained-release manner. On the other hand, the release of MP from FPEGMP-2 nanocomposite was much slower than that from the physical mixture, indicating a controlled release property of the latter.

It was found that the release rate of MP from FPEGMP-2 is affected by the acidity of the media. Due to the “burst effect” [49] and other mechanisms, the release behavior of MP shows a fast release at the beginning, 67% for the first 4 hours, followed by a slower stage of 85% for the second 74 hours at pH 4.8 (Figure 6(B)). At pH 7.4, the release rates of MP are slower than that at pH 4.8 and the maximum percentage release reaches about 56% at about 92 hours (Figure 6(A)). Therefore, the result reveals that the FPEGMP-2 nanocomposite shows a good potential to be used as a drug delivery with controlled release property.

**Figure 7:**
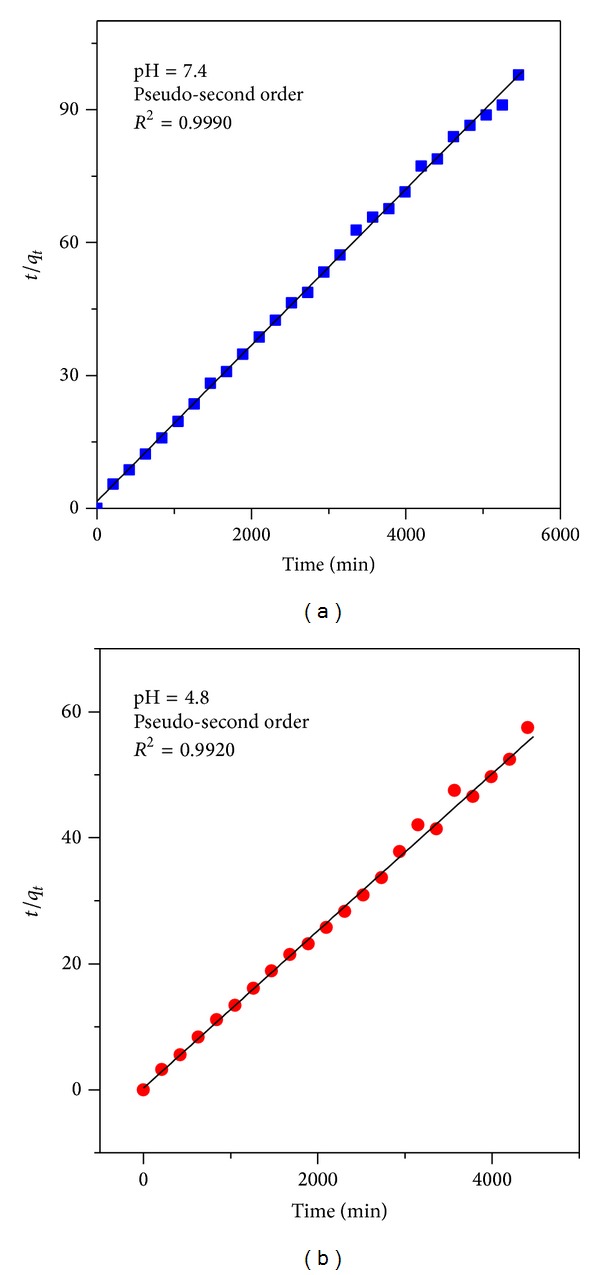
Fitting the data of MP release from FPEGMP-2 nanocomposite into phosphate buffered solution to the pseudo-second order kinetics for pH 7.4 (a) and pH 4.8 (b).

In order to obtain more insight into the mechanism of release of MP from FPEGMP-2 nanocomposite, three different kinetic models were used to fit the release data. The pseudo-first order kinetic equation [50] (ln⁡(*q*_*e*_ − *q*_*t*_) = ln⁡*q*_*e*_ − *k*_1_ *t*) represents the release of MP from FPEGMP-2 nanocomposite and the decomposition rate depends on the amount of MP in the FPEGMP-2 nanocomposite. The other two kinetic models can be described by pseudo-second order model [51] which can be expressed in the form of (*t*/*q*_*t*_ = 1/*k*_2_ *q*_*e*_^2^ + *t*/*q*_*e*_) and the parabolic diffusion model which can be represented as [52] (1 − *M*_*t*_/*M*_0_)/*t* = *kt* ^−0.5^ + *b* equations. In pseudo-first order equation and the pseudo-second order kinetic model, the *q*_*e*_ and *q*_*t*_ are the equilibrium release rate and the release rate at time *t*, respectively. Also *k*, in all three models, is a constant and corresponding to the release amount. The *M*_0_ and *M*_*t*_ in parabolic equation are the drug content remained in FPEGMP-2 nanocomposite at release time 0 and *t*, respectively. Through the basis of these kinetic models, as mentioned earlier for the release kinetic data, it was found that the pseudo-second order kinetic model can be more satisfactory in order to describe the release behavior of 6-mercaptopurine from FPEGMP-2 nanocomposites compared to the other models used in this work (Figures 7(a) and 7(b) and Table 2).

### 5.7. *In Vitro* Bioassay

**Figure 8:**
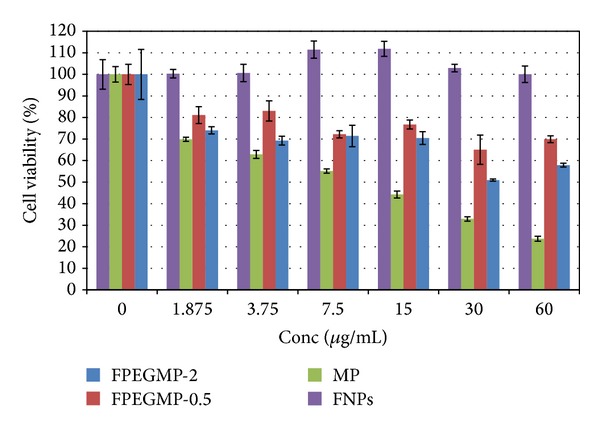
Showing* in vitro* cytotoxicity studies of WEHI-3B cells after 48 hours of exposure to free MP, iron oxide nanoparticles (FNPs), FPEGMP-0.5, and FPEGMP-2 nanocomposites. The two nanocomposites (FPEGMP-0.5 and FPEGMP-2) and pure MP showed continuous cell viability, decreased with each increase in dose. Their IC_50_ values were found to be 10 ± 0.5 *μ*g/mL, 30 ± 3.0* µ*g/mL, and 6.0 ± 2.0* µ*g/mL for MP, FPEGMP-2, and FPEGMP-0.5, respectively, as obtained from the graph and calculated via regression analysis.

Figure 8 shows a dose-dependent effect of FNPs, MP, FPEGMP-0.5, and FPEGMP-2 nanocomposites. Pure MP showed a higher anticancer effect on the leukemic cell line compared to the other two nanocomposites (FPEGMP-0.5 and FPEGMP-2) within the tested doses. The uncoated iron oxide nanoparticles demonstrated sustained leukemic cell viability even in the presence of increased concentration. This finding is similar to a previous study done on both normal and cancerous cell lines exposed to iron oxide nanoparticles up to 30 *μ*g/mL concentration, where more than 80% of the cell survived the nanoparticles treatment [1, 53].

The two nanocomposites, FPEGMP-0.5 and FPEGMP-2, showed lower anticancer activity in almost all the concentration tested compared to pure MP. However, in our previous study [1], we reported enhanced anticancer activity of the 6-mercaptopurine on the same leukemic cell line after coating with FNPs-chitosan. This finding concurred with other previous work [54], where PVP-coated silver nanoparticles induced greater cytotoxicity than citrate-coated particles. Surface coating of nanoparticles has been shown to affect affinity for cell surface adhesion as well as dissolution [54]. In another related study chitosan-coated magnetic nanoparticles showed higher cell capture rate than starch coating [55]. The capture rate on fibro sarcoma cell lines was found to be 73.4 and 64.1% for chitosan and starch, respectively.

Anticancer activity of FPEGMP-2 nanocomposite was found to be slightly higher than FPEGMP-0.5 in a dose-dependent manner on the leukemic cell lines (Figure 8). This may be attributed to the differences in percentage of 6-mercaptopurine between the two nanocomposites. Thus, choice of coating material as well as percentage loading of active agent on a nanocarrier was shown to affect the activity of the resulting materials.

## 6. Conclusion

The iron oxide nanoparticles prepared via coprecipitation method are of magnetite material with the mean size of 10 nm. Similarly, the PEG-coated nanoparticles, FPEGMP-2, are composed of pure magnetite core with particle mean size of 11 nm. The attachment of PEG-MP in the latter onto the surface of the former was supported by FTIR findings. Vibrating sample magnetometer studies confirm the superparamagnetic properties of iron oxide nanoparticles (FNPs) and the FPEGMP-2 nanocomposite. The thermal stability of the resulting nanocomposite (FPEGMP-2) compared to the pure drug (MP) was found to improve after the coating process. The release behavior of MP from FPEGMP-2 nanocomposite into phosphate buffered solution was found to be of controlled manner with release percentage of about 60% and 97% when exposed to pH 7.4 and 4.8, respectively. It was found that FPEGMP-2 demonstrated slightly higher anticancer activity on leukemic WEHI-3B cell lines than the FPEGMP-0.5 nanocomposite in a dose-dependent manner. The uncoated FNPs demonstrated sustained leukemic cell viability even in the presence of increased concentration. This may be due to the differences in percentage of 6-mercaptopurine between these two nanocomposites (FPEGMP-0.5 and FPEGMP-2). Therefore, the choice of coating material as well as percentage loading of the active agent on a nanocarrier was shown to affect the cytotoxicity activity.

## Figures and Tables

**Table 1:** Magnetic properties of FNPs and FPEGMP-2 nanocomposite.

Samples	*M*_*S*_ (emu/g)	*M*_*r*_ (emu/g)	*Hc* (G)
FNPs	54.641	1.2314	20.655
FPEGMP-2	38.635	0.5860	23.220

**Table 2:** Correlation coefficient, rate constant, and half-time obtained by fitting the data of the release of MP from FPEGMP-2 nanocomposite into phosphate-buffered solution at pH 4.8 and 7.4.

Aqueous solution	Saturated release %	*R* ^2^			Rate constant (*k*)^a^(mg/min)	*t*_1/2_^a^ (min)
		Pseudo-first order	Pseudo-second order	Parabolic diffusion		
pH 7.4	59.6	0.4017	0.9990	0.4876	1.89 × 10^−4^	93
pH 4.8	97.2	0.9168	0.9950	0.9567	5.59 × 10^−4^	22

^a^Estimated using pseudo-second order kinetics.
